# Erythropoietin: a multimodal neuroprotective agent

**DOI:** 10.1186/2040-7378-1-4

**Published:** 2009-10-21

**Authors:** Nadiya Byts, Anna-Leena Sirén

**Affiliations:** 1 University of Würzburg, Department of Neurosurgery, Würzburg, Germany

## Abstract

The tissue protective functions of the hematopoietic growth factor erythropoietin (EPO) are independent of its action on erythropoiesis. EPO and its receptors (EPOR) are expressed in multiple brain cells during brain development and upregulated in the adult brain after injury. Peripherally administered EPO crosses the blood-brain barrier and activates in the brain anti-apoptotic, anti-oxidant and anti-inflammatory signaling in neurons, glial and cerebrovascular endothelial cells and stimulates angiogenesis and neurogenesis. These mechanisms underlie its potent tissue protective effects in experimental models of stroke, cerebral hemorrhage, traumatic brain injury, neuroinflammatory and neurodegenerative disease. The preclinical data in support of the use of EPO in brain disease have already been translated to first clinical pilot studies with encouraging results with the use of EPO as a neuroprotective agent.

## The cytokine erythropoetin (EPO)

The cytokine erythropoietin (EPO) is a 34 kD glycoprotein which was originally described to stimulate erythropoiesis. EPO supports the proliferation and differentiation of erythroid progenitor cells and is critical for their survival [1]. The main site of EPO production is fetal liver and adult kidney [1]. Mice deficient for EPO or EPO receptor (EPOR) genes die at embryonic day 13 (E13) because of severe anemia caused by deficiency in definitive erythropoiesis [2-4]. Over the last decade it has become clear that EPO acts as growth and survival factors for multiple tissues expressing the EPO receptor [1]. The number of described targets of EPO action continues to grow.

## EPO Receptor (EPOR)

EPO acts by binding to its specific transmembrane receptor (EPOR). EPOR belongs to the single-chain cytokine type I receptor family [5]. These receptors are characterized by an extracellular N-terminal domain with conserved cysteines and a WSXWS-motif, a single hydrophobic transmembrane segment and a cytosolic domain with no intrinsic kinase activity [5]. Two transmembrane EPOR molecules form a homodimer that binds one EPO molecule leading to a conformational change and tight bonding of the two EPOR monomers which in turn activate two Janus family tyrosine kinase 2 (JAK2) molecules which associate with cytoplasmic domain of the EPOR. Once activated, JAK2 phosphorylates distal parts of receptors which subsequently serve as docking sites for downstream signaling molecules. Multiple signal transduction pathways are activated downstream of EPOR/JAK2 [1,5]. In neurons these include signal transducers and activators of transcription (Stat), phosphatidylinositol 3-kinase (PI3K)/Akt, Ras/extracellular signal regulated kinase (ERK1/2), nuclear factor-kappa-B (NF-κB), and calcium [6-8]. Best investigated from these are PI3K/Akt and Ras-MAPK pathways, both of which are important for the antiapoptotic and trophic effects of EPO [8-18]. NFκB pathway plays a role in antiapoptotic activity of EPO in neurons [7,19,20] as well as in EPO-mediated propagation of neural stem cells [21]. In addition EPO by activating phospholipase Cγ modulates intracellular calcium concentration, electrical activity and neurotransmitter release in PC12 cells [22-24] as well as in hippocampal neurons and brain slices [25,26]. The role of the Stat transcriptional factors in regard to EPO effects in the neural cells remains unclear; EPO induces phosphorylation of Stat5 in neurons [8,19,27], neural stem cells [21] and neuroblastoma cells [17,28]. Using primary hippocampal neurons from Stat5a/b knock-out mice we have recently shown that the presence of functional Stat5 is required for the trophic but not for the protective effect of EPO against glutamate-induced toxicity [9].

EPO signaling is terminated by activation of phosphatases which dephosphorylate JAK2. The ligand-receptor complex is then internalized and degraded by the proteasome [1,5]. In hematopoietic cell lines 60% of the internalized EPO is re-secreted [29].

## The brain EPO/EPOR system

mRNA and protein of EPO and EPOR are detected in brain (hippocampus, internal capsule, cortex, midbrain), as well as *in vitro *in neurons, astrocytes, oligodendrocytes, microglia and cerebral endothelial cells [24,30-44] suggesting that this factor can function in the brain in a paracrine and/or autocrine manner. In the developing mouse brain expression of EPO and EPOR peaks during midgestation and decreases to adult levels in late gestation [43]. Brain specific ablation of EPOR leads to deficits in neural cell proliferation and neuronal survival in the embryonic brain and in post-stroke neurogenesis in the adult brain [45,46]. Expression of EPO and EPOR in the adult brain is stress-responsive and is regulated by oxygen supply: Both receptor and ligand expression is upregulated after hypoxia or ischemia [36,42,43,47-50]. Other stimuli such as hypoglycemia, insulin release, reactive oxygen species and insulin-like growth factor activate hypoxia-inducible factor and lead to increased expression of EPO [51,52]. Proinflammatory cytokines downregulate expression of EPO mRNA but increase that of EPOR in astrocytes [34]

Based on the loss of some of the tissue protective effects of EPO and its non-hematopoietic derivative, the carbamylated EPO (CEPO) in mice lacking the common β chain shared by the members of the IL-3 receptor family, Brines and Cerami have proposed that the cytoprotective effects of EPO and CEPO are mediated by a heteromeric receptor complex comprised of one EPOR subunit and a dimer of the common β chain [6,53]. Immunoreactivity of the common β chain has been detected in brain tissue with a pattern reminiscent to that of the classical EPOR [53]. Furthermore, the common β chain can be coimmunoprecipitated with EPOR antibodies from the P19 embryonal carcinoma cells [53], but the existence of the proposed heteromeric receptor structure in primary cells or tissues has yet to be directly proven. In a recent study no expression of the common β subunit was detected in neuronal PC-12 cells even if EPO rescued these cells from staurosporine-induced apoptosis [28]. Interestingly, the classical EPOR is required for EPO-stimulated neuronal differentiation and survival but not for neurogenesis induced by CEPO suggesting that differential receptor interactions mediate the effects of EPO and CEPO in brain cells [28,54].

## Neuroprotection by EPO is independent of hematopoesis

The neuroprotective actions of EPO can be separated from its hematopoietic actions, a fact that is of value for therapeutic applications where the increase in red cell mass is not desired. EPO and EPO derivatives are directly neuroprotective in cell culture models (see below) and after direct application into the brain [36,55,56]. Moreover, CEPO and other EPO derivatives which do not bind to EPOR in myeloid cells and thus lack hematopoietic activity display potent tissue protective activities [57-59]. Expression of EPO and the classical EPOR in brain cells is induced by hypoxic-ischemic stress and contributes to ischemic tolerance [14,24,32,37,41,49,50,60-66] while neutralization of the brain endogenous EPO augments ischemic damage [56]. Brain-specific genetic ablation of the classical EPOR impairs post-stroke neurogenesis and neuronal survival [45,46] whereas transgenic brain specific overexpression of human EPO is associated with reductions in postischemic infarct volume, brain swelling and functional deficits in a transient stroke model [16].

## Multimodal neuroprotective profile

**Figure 1:**
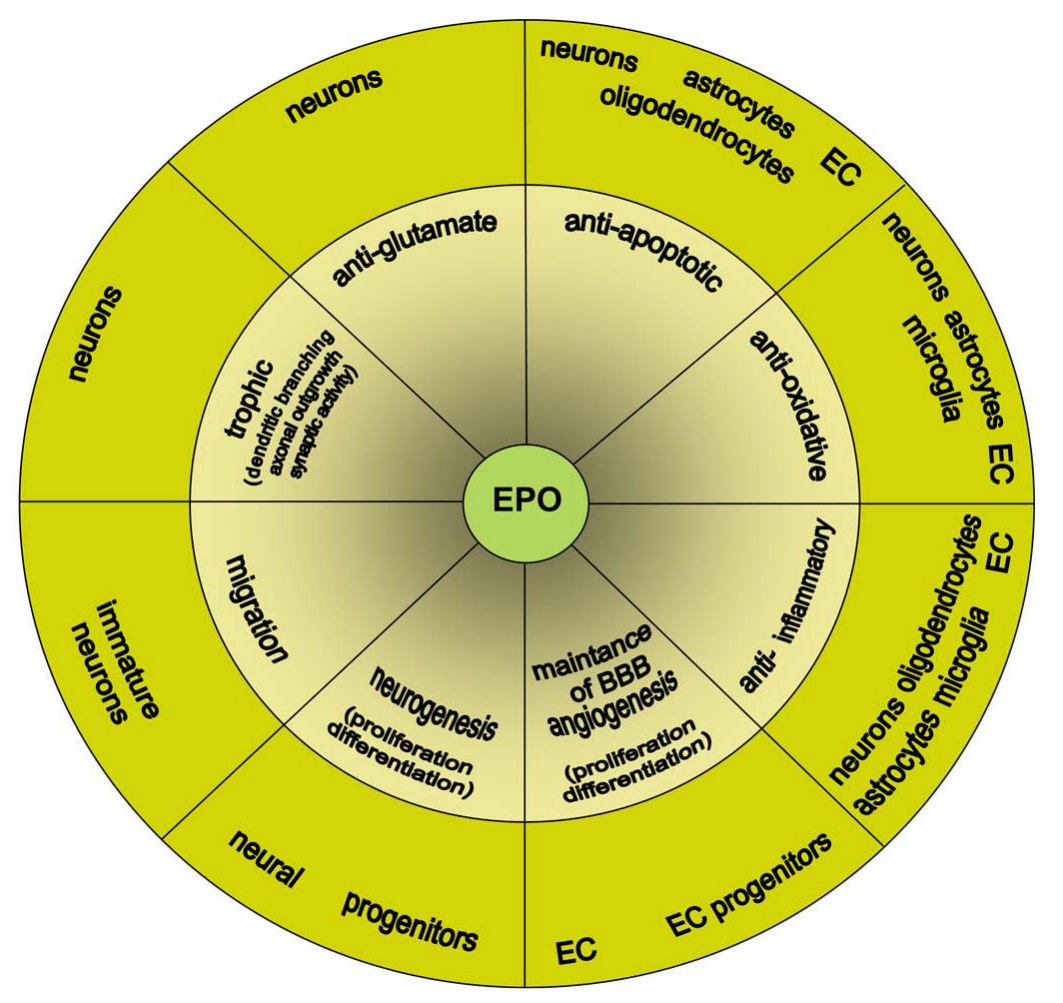
**Multimodal neuroprotective profile of erythropoietin (EPO)**. BBB - blood brain barrier; EC - endothelial cells.

EPO has been reported to induce a broad range of cellular responses in the brain directed to protect and repair tissue damage (Figure 1). EPO is neuroprotective in a variety of hypoxic, hypoglycemic, and excitotoxic *in vitro *models [7,8,10,14,17,18,20,21,42,45,50,57,67-72]. A fundamental mechanism of EPO-induced neuroprotection in cultured neurons is its ability to inhibit apoptosis reducing both DNA damage and cell membrane asymmetry [7,8,10,14,17,18,20,21,42,45,50,57,67-72]. Necrotic cell death (for example, after glutamate exposure) is also be attenuated by EPO [9,69,73]. Why astroglial cultures are protected by EPO from nitric oxide- and staurosporine-induced death but not from arsenic trioxide- or glutamate-induced toxicity is not fully understood [73,74].

Another tissue-protective mechanism of EPO is its ability to protect cells against oxidative damage [75,76]. EPO inhibits lipid peroxidation by increasing the activities of cytosolic antioxidant enzymes such as superoxide dismutase and glutathione peroxidase [77-79].

EPO attenuates inflammation by reducing reactive astrocytosis and microglia activation and by inhibiting immune cells recruitment into the injured area [47,58,59,70,80-85]. In cerebrovascular endothelial cell cultures EPO down-regulates TNF-α-induced gene expression of interleukin-6 (IL-6), IL-1beta, CXCR4, and IL-1alpha [86]. It also directly counteracts interferon-γ- and lipopolyssaccharide-induced cytotoxicity in oligodendrocytes, preserves white matter [87] and reduces TNF-α release and its effects in cultured Schwann cells [88].

EPO protects vascular integrity and stimulates angiogenesis [89-92]. It preserves blood-brain barrier integrity during injury by restoring expression of tight junction proteins [90,93,94], by reducing vascular inflammation [95] and reactive free radical expression [90,93,94,96]. In vasculogenesis EPO stimulates proliferation of endothelial precursor cells, production of matrix metalloproteinase-2, migration of endothelial cells into vascular sites and formation of capillary tubes [90-92,97,98]. EPO displays direct antiapoptotic activity in cerebral endothelial cells during oxidative stress and ischemic injury as well [91]. Stimulation of endothelial nitric oxide synthase (eNOS) activity has been shown to contribute to the improvements by EPO after experimental cerebral hemorrhage [99-101]. Interestingly, plasma and tissue levels of NO are markedly increased in transgenic rats overexpressing EPO [102] whereas the vascular protection by EPO is abolished in eNOS-deficient mice [103].

EPO promotes differentiation towards neurons in several neuroblastoma cell lines, in neural stem cell cultures derived from both embryonic and adult neuronal germinal zones, as well as in embryonic neural progenitor-cell cultures [21,45,46,54,89,104-108]. Neuronal differentiation of adult neural progenitor cell by EPO seem to require activation of the sonic hedgehog signaling and up-regulation of suppressor of cytokine signaling-2 (SOCS2) [54,105]. EPO increases proliferation of oligodendrocyte progenitors and promotes differentiation of oligodendrocytes in culture [33,34]. EPOR^-/- ^fetuses exhibit increased apoptosis in the brain and a reduction in the number of neural progenitor cells, as well as increased sensitivity to hypoxia prior to significant anaemia or heart defects in the embryo proper [42,45,46]. Moreover, adult mice that lack EPOR in the brain have significantly reduced neurogenesis in the subventricular zone and demonstrate impaired migration of precursors into infracted cortex [46]. Nevertheless, expression of EPO or EPOR on neural cells is not indispensable for brain development [42,45,46].

The reported neurotrophic effects of EPO include the ability to stimulate axonal regrowth, neurite formation, dendritic sprouting, electrical activity and modulate intracellular calcium and neurotransmitter synthesis and release [9,13,22,23,25,26,46,109-113]. A recent study demonstrated a calcium sensitive activation of cAMP response element binding protein (CREB) and induction of brain-derived neurotrophic factor (BDNF) gene expression by EPO in primary hippocampal neurons [25]. In rat hippocampal slices, EPO improved synaptic transmission during and following oxygen and glucose deprivation [13]. However, it has not been directly shown that EPO induces formation of new synapses.

## Animal Studies

The preclinical data in support of the use of EPO in human brain disease have explosively increased since the first discovery of its neuroprotective action. In particular, the preclinical evidence in support for testing EPO in human acute ischemic stroke fulfills most of the STAIR criteria [114] such as testing by several laboratories using both temporary and permanent stroke models, post-treatment at several doses and exploration of therapeutic window, characterization of pharmacokinetic profile in respect to blood-brain-barrier penetration after peripheral administration, measurement of histological and functional outcome with prolonged survival.

## Cerebral ischemia

The *in vivo *potential of EPO to protect neurons against ischemic neuronal damage was first provided by the Sasaki group. Their landmark finding was that application of recombinant human (rh)EPO directly into the cerebral ventricles of gerbils prevented ischemia-induced learning disabilities and protected hippocampal pyramidal CA1 neurons from lethal ischemia while neutralization of the endogenous brain EPO by infusions of soluble EPOR before a nonlethal mild ischemia induced neuronal death [56]. Since the circulating EPO, as a large, highly glycosylated negatively charged molecule, was thought not to cross the blood-brain-barrier [91,115,116], the early studies used direct intracerebroventricular route of administration of EPO to demonstrate its potent tissue protective activity in focal and global models of cerebral ischemia [36,55,56]. The first evidence for a neuroprotective effect of EPO by peripheral route of administration was provided by Brines et al. (2000) who demonstrated in a focal stroke model reduction of infarct volumes by intraperitoneally applied high dose rhEPO (5000 U/kg) up to 6 h after reperfusion. Immunohistochemical detection of biotinylated rhEPO 5 hours after its intraperitoneal injection at the therapeutically effective dose (5000 U/kg) further provided evidence that EPO can cross the blood-brain barrier [117]. Studies in several species including man have confirmed the ability for high dose EPO to cross the blood-brain barrier in therapeutic effective concentrations [118-122]. To date EPO and its derivatives have shown to reduce histological damage and improve functional outcome when given as intraperitoneal or even intranasal post-treatment after experimental stroke [57-59,70,85,89,123], global cerebral ischemia [124], neonatal stroke and hypoxia-ischemia [107,125-128]. For example, a comprehensive dosing study using post-treatment with EPO and CEPO starting at 6 h after an embolic middle cerebral artery occlusion in rats demonstrated reduction of functional deficits and infarct volume up to 28 days models of middle cerebral artery occlusion [59]. Induction of EPO and its intracellular signaling intermediates represents a significant component of tolerance induced by ischemic or hypoxic preconditioning [60-62,65]. Here activation of the antiapoptotic and anti-inflammatory signaling seems to play a major role in the EPO-induced neuroprotection [14,19,129].

## Cerebral hemorrhage

Post-treatment with EPO starting at 2 h after induction of intracerebral hemorrhage (ICH) by intraparenchymal injections of collagenase or autologous blood dose-dependently reduced volume of hemorrhage, brain edema, perihematomal apoptosis and inflammation in a rat model [99]. Functional recovery was faster and more efficient in the EPO-treated group and was associated with reduction in hemispheric brain atrophy 5 weeks after the induction of ICH [99]. Cerebral vasospasm and ischemic brain damage after subarachnoid hemorrhage (SAH) by autologous blood injections into the cisterna magna in rabbits are reduced by EPO administered either by intraperitoneal injections of rhEPO or by delivery of adenoviral vectors encoding the human EPO into cisterna magna immediately after induction of SAH [130-133]. Mortality and functional deficits 3 days after induction of SAH were reduced in EPO treated rabbits [130-132]. In a rat model of SAH, the impaired autoregulatory response of cerebral blood flow to intravenous noradrenaline was restored by a single subcutaneous bolus of EPO [134].

## Traumatic brain and spinal cord injury

Administration of EPO and EPO-analogs in experimental models of traumatic brain and spinal cord injury leads to morphological, functional and cognitive recovery that can be attributed to a multitude of cytoprotective mechanisms including inhibition of apoptosis, anti-inflammatory and anti-oxidant actions, restoration of blood-brain barrier integrity, stimulation of neurogenesis and angiogenesis [67,95,96,104,117,135-146]. Induction of EPO and its protective down stream signaling via Akt seems also to account for the protective effect of heat acclimation stress in a closed head injury model [147,148]. Brain edema after experimental brain injury can effectively be attenuated by post-treatment with EPO [95,138,140]. A reduction of cytotoxic and vasogenic edema may be anticipated based on the direct actions of EPO on glutamate release [112] and on the endothelial barrier function (see above). It is not clear to date which from the panoply of neurorestorative effects of EPO are responsible for the long-term prevention of trauma-induced brain atrophy, cognitive and neurobehavioral dysfunction [104,135-137,146]. In this context it is interesting to note that chronic peripheral administration of EPO has been reported to improve spatial memory function and cognitive functioning in the context of an aversion task also in healthy mice [119,149]. Improved hippocampal functioning after a single intravenous bolus of EPO was recently shown in a study using functional magnetic resonance imaging in healthy human volunteers [150].

## Degeneration & neuroinflammation

EPO and its analogs offer protection also in models of neurodegenerative and neuroinflammatory disease. In experimental autoimmunencephalitis (EAE), an animal model for multiple sclerosis (MS), treatment with EPO and EPO analogs can improve functional recovery, reduce tissue damage, inflammatory responses and blood-brain barrier leakage [47,80-84,117]. Beneficial effects of EPO have also been reported in models of peripheral axonal nerve injury, injury-induced Wallerian degeneration and HIV-associated sensory neuropathy [88,151,152]. Here, the anti-cytokine, anti-apoptotic, anti-oxidative and trophic effects on both neurons and oligodendrocyte progenitor cells by EPO seem to play an important role in reducing inflammation and preserving myelination and neuronal function [35,47,80-84,86-88,151].

Chronic neurodegeneration might also be a target for EPO therapy as EPO and its analogs can counteract degenerative processes in experimental models of Parkinson disease and amyotrophic lateral sclerosis (ALS) by inducing anti-oxidant enzymes, inhibiting apoptosis and stimulating axonal regeneration [78,153-155]. EPO improved graft survival of embryonic ventral mesencephalic dopamine neurons when transplanted into the striatum of 6-hydroxy-dopamine lesioned rats [156]. However, not all degenerative diseases seem to respond to EPO therapy [157].

## Translation to human brain disease

EPO and its receptor are abundantly expressed in the developing human brain decreasing to a weak constitutive expression in the adult [30,39,41,66,119,158]. Hypoxia rapidly induces expression of brain EPO as evidenced by the increased expression of EPO in cerebrospinal fluid (CSF) or postmortem brain tissue in humans with traumatic brain injury, SAH, stroke and hypoxia [31,41,66,121,159,160]. Expression of EPOR has also been detected on myelin sheath of radicular nerves and in the epineurial blood vessels of sural nerves in the human peripheral nervous system [161,162]. Measurements of endogenous levels of EPO in CSF of patients with neurodegenerative diseases has revealed EPO in CSF of patients with ALS to be lower than in controls whereas patients with Alzheimer disease (AD) or vascular dementia had EPO levels comparable to control persons [163,164]. Astrocytic EPOR immunoreactivity in postmortem hippocampus and temporal cortex from subjects with AD or chronic schizophrenia has been reported to be increased as compared to age-matched control brains [119,158]. In AD, however, no association of EPOR-positive astrocytes was found with summary measures of global cognition or AD pathology [158].

Millions of patients have already been receiving EPO as a highly efficacious and safe treatment for anemia [1]. Indeed, the first proof-of-concept study on use of EPO in human acute ischemic stroke has already demonstrated that treatment of stroke patients with intravenous high dose EPO is not only well tolerated but is associated with improvement in clinical outcome at 30 days [120]. Encouraging results with the use of EPO as a neuroprotective agent have been recently reported in clinical pilot studies after out-of-hospital cardiac arrest [165], ureamia-associated peripheral neuropathy [166], chronic schizophrenia [167] and MS [145]. A small pilot study to probe the safety and efficacy of EPO in SAH was recently preliminarily terminated with no conclusive results because of low recruitment rate [168]. To date a randomized multicenter double blinded placebo-controlled clinical trial in acute ischemic stroke is running (for details see http://www.epo-study.de) reflecting the beginning of exploration of EPO and its analogs for clinical neuroprotection in large phase II/III setting.

## Competing interests

The authors declare that they have no competing interests.

## Authors' contributions

NB drafted the manuscript and designed the Figure. ALS corrected and wrote the final manuscript. Both authors read and approved the final manuscript.
